# Label-free flow cytometry of rare circulating tumor cell clusters in whole blood

**DOI:** 10.1038/s41598-022-14003-5

**Published:** 2022-06-24

**Authors:** Nilay Vora, Prashant Shekhar, Michael Esmail, Abani Patra, Irene Georgakoudi

**Affiliations:** 1 Department of Biomedical Engineering, Tufts University, Medford, MA 02155 USA; 2 Department of Mathematics, Embry-Riddle Aeronautical University, Daytona Beach, FL 32114 USA; 3 Tufts Comparative Medicine Services, Tufts University, Medford, MA 02155 USA; 4 Department of Computer Science, Tufts University, Medford, MA 02155 USA

**Keywords:** Cancer, Breast cancer, Cancer imaging, Metastasis, Tumour biomarkers, Biomedical engineering, Computer science, Imaging and sensing

## Abstract

Circulating tumor cell clusters (CTCCs) are rare cellular events found in the blood stream of metastatic tumor patients. Despite their scarcity, they represent an increased risk for metastasis. Label-free detection methods of these events remain primarily limited to in vitro microfluidic platforms. Here, we expand on the use of confocal backscatter and fluorescence flow cytometry (BSFC) for label-free detection of CTCCs in whole blood using machine learning for peak detection/classification. BSFC uses a custom-built flow cytometer with three excitation wavelengths (405 nm, 488 nm, and 633 nm) and five detectors to detect CTCCs in whole blood based on corresponding scattering and fluorescence signals. In this study, detection of CTCC-associated GFP fluorescence is used as the ground truth to assess the accuracy of endogenous back-scattered light-based CTCC detection in whole blood. Using a machine learning model for peak detection/classification, we demonstrated that the combined use of backscattered signals at the three wavelengths enable detection of ~ 93% of all CTCCs larger than two cells with a purity of > 82% and an overall accuracy of > 95%. The high level of performance established through BSFC and machine learning demonstrates the potential for label-free detection and monitoring of CTCCs in whole blood. Further developments of label-free BSFC to enhance throughput could lead to important applications in the isolation of CTCCs in whole blood with minimal disruption and ultimately their detection in vivo.

## Introduction

Tumor growth from a localized to a distant or metastatic state in cancer patients significantly reduces the five-year survival rate^1^. Rare circulating tumor cells (CTCs) and rarer circulating tumor cell clusters (CTCCs) are considered primary vehicles of metastatic tumor formation^2,3^. CTCCs, in particular, are 23–50 times more likely to lead to a metastasis in comparison to individual CTCs^4^. Since their discovery in the 1970s, little progress has been made to address the clinical value and metastatic advantage of CTCCs^5,6^. Thus, there is an interest in detecting and isolating CTCCs to further understand their increased metastatic potential and to develop new treatments to target CTCCs. A key limitation in detection and isolation of CTCCs is their prevalence in the blood stream of patients, with CTCCs occurring at a rate of less than 3.75 events/7.5 mL of blood in metastatic tumor patients^7^. In comparison, there are 37.5 billion red blood cells (RBCs) and 56.25 million white blood cells (WBCs) typically present in 7.5 mL of whole blood^8^.

To date, the only FDA-approved platform for CTC detection is CellSearch, which uses magnetic-activated cell sorting for positive detection and isolation of CTCs and CTCCs^2,7,9,10^. While CellSearch has become the diagnostic standard for CTCs, the platform’s reliance on targeting the epithelial cell adhesion molecule (which is not always present on all CTCs and CTCCs), inability to detect CTCs in ~ 30–35% of metastatic breast cancer patients, and poor correlation with prognosis limits its application^2,9,11,12^.

Recent work in microfluidic technology has provided innovative methods for CTC and CTCC capture, such as the deterministic lateral displacement (DLD) chip and the non-equilibrium inertial separation array (NISA-XL) chip^13,14^. These platforms demonstrate high levels of in vitro sorting of CTCCs from whole blood, improving the detection sensitivity in comparison to CellSearch. However, these platforms rely on blood draws, which limit interrogation volume and can lead to over or underestimation of events.

To overcome this, several groups have explored in vivo flow cytometry (IVFC) to monitor circulating events such as CTCs and CTCCs in vivo^15–21^*.* A majority of these techniques utilize exogeneous fluorescence-based contrast to detect CTCCs, limiting their clinical translatability, at least in the short term. The only IVFC technique to collect label-free data in vivo for CTCC detection to our knowledge is the photoacoustic flow cytometer designed by Galanzha et al.^21^. This platform successfully demonstrated detection of cluster events in vivo in humans; however, its application for cancer cell detection is limited to melanoma patients, as the technology relies on the strong absorbance of melanoma cells to detect their presence in flow^21^. There are also a limited number of studies that have detected CTCs in a label-free manner based on endogenous fluorescence and coherent anti-stokes Raman; however, none of these methods have been used for CTCCs^18^.

Previously, our group demonstrated through in vitro label-free flow cytometry that CTCCs have unique scattering signatures in comparison to white blood cells^9^. In this study, we build on our work with backscatter flow cytometry (BSFC) to assess the potential of detecting unique endogenous scattering signatures of CTCCs in whole blood. To meet this objective, fresh whole blood samples from rodents were spiked with GFP-expressing CTCCs and flowed through microfluidic channels. Light scattering and fluorescence data collected in these flow cytometry studies were used to train and evaluate the performance of a machine learning algorithm, relying on the GFP-detected CTCC peaks as ground truth. Validation and testing of this algorithm demonstrate that unique endogenous scattering CTCC signatures can be identified to enable label-free detection of CTCCs in whole blood with high accuracy.

## Results

**Figure 1:**
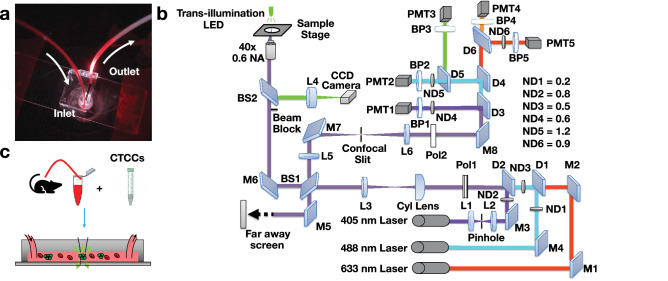
(**a**) Microfluidic device. (**b**) Flow cytometer schematic. *D* dichroic, *L* lens, *M* mirror, *BP* bandpass filter, *BS* beam splitter, *PMT* photomultiplier tube, *Pol* polarizer, *CAM* CCD camera, *ND* neutral density filter, *Cyl Lens* cylindrical lens. (**c**) Schematic of experimental design for detection of CTCCs in whole blood flowed through a 30 × 30 μm^2^ microfluidic channel.

**Figure 2:**
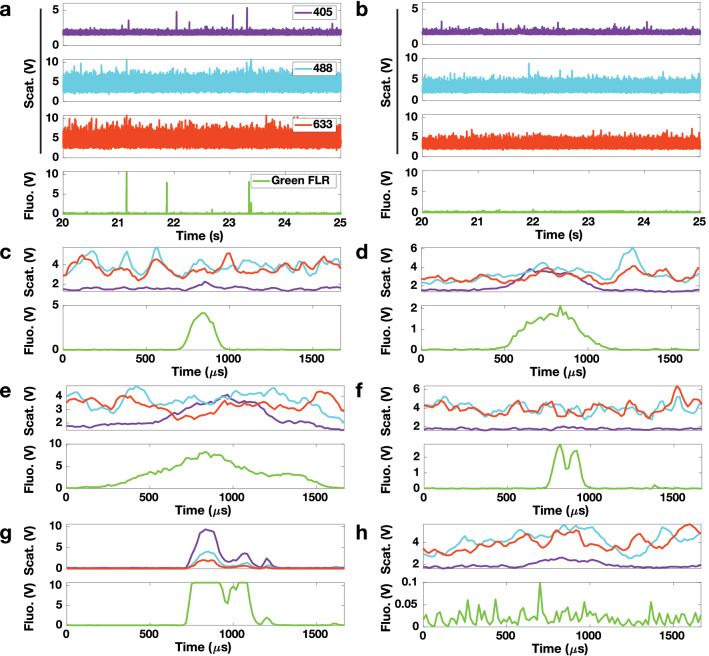
Raw data traces of CTCs/CTCCs and false positive events detected in sample. (**a**) A 5-s-long time trace of scattering and fluorescence signals collected from a whole blood sample spiked with CTCs/CTCCs and (**b**) an unspiked whole blood sample from the same animal. (**c**) A single CTC with a narrow FWHM (11 points) suggesting this belongs to an individual cell. (**d**) A small CTCC with a peak width of 24 points representing a small cluster 3–4 cells in size. (**e**) A large CTCC with a peak width of 30 points representing a large cluster 6+ cells in size. These events are labeled as CTCs/CTCCs based on their broad peak widths and green fluorescence signal, which is being used as our ground truth label. (**f**) Shows a peak event with a strong green fluorescence signal with no clear scattering signal. (**g**) A local trace from a 3–4 cell cluster with a peak width of 24 points from flowing CTCCs in cell growth media. (**h**) A local trace from an event that is incorrectly labeled as a CTCC by the initial peak detection algorithm.

Whole blood samples from non-tumor bearing rodents were spiked with CTCCs and flowed through the 30 × 30 μm^2^ channels of a microfluidic device (Fig. 1a,c, Supplementary Fig. S2 online). To interrogate the sample, three laser light sources were used to irradiate the sample: a 405 nm, 488 nm, and 633 nm laser (Fig. 1b). The illumination beam was focused into a sharp slit using a cylindrical lens, which was re-imaged by an objective lens to traverse the microfluidic channel (Fig. 1c). Backscattered and fluorescence light was collected by the same objective and directed to five photomultiplier tubes. Scattered signals from the three illumination wavelengths were detected by PMT1, PMT2, and PMT5; additionally, green (500–550 nm) and red (650–690 nm) fluorescence signals were collected by PMT3 and PMT4, respectively (Fig. 1b). CTCCs were engineered to express green fluorescence protein (GFP) for ground truth comparison. GFP signal was collected by PMT3. Sample data traces from flowing whole blood specimens spiked with GFP labeled CTCs and various size CTCCs are shown in Fig. 2. As expected, the strong GFP signal even from single cells yielded high signal to noise ratio (SNR) peaks (green traces). Sample whole blood time traces collected from the same rat are shown, with one sample being spiked with cancer cells (Fig. 2a) and the other flowed as collected (Fig. 2b). Since the flow speed of the sample was known, the width of the peaks could be associated with the cross-sectional diameter of the object from which each peak originated. Based on this width, the peaks shown were attributed to a CTC (Fig. 2c), and CTCCs of 3–4 or more than 6 cells (Fig. 2d,e, respectively). While many GFP peaks corresponded to similar peak features detected in two or three of the scattering channels (Fig. 2c–e), some GFP peaks did not yield any distinct detectable features in any of the scattering channels (Fig. 2f). An observed limitation of detecting CTCCs in whole blood was the increased background scattering signal. A sample 3–4 cell CTCC time trace collected from CTCCs in cell growth media (Fig. 2g) demonstrates the impact of blood scattering on SNR in comparison to a similarly sized cluster in whole blood (Fig. 2d). In addition, there were several instances where the scattering channels exhibited changes that appeared similar to those observed in Fig. 2c–e but did not correspond to a peak detected in the GFP channel (Fig. 2h). We refer to the latter peaks as non-CTCC (NC) peaks.

**Figure 3:**
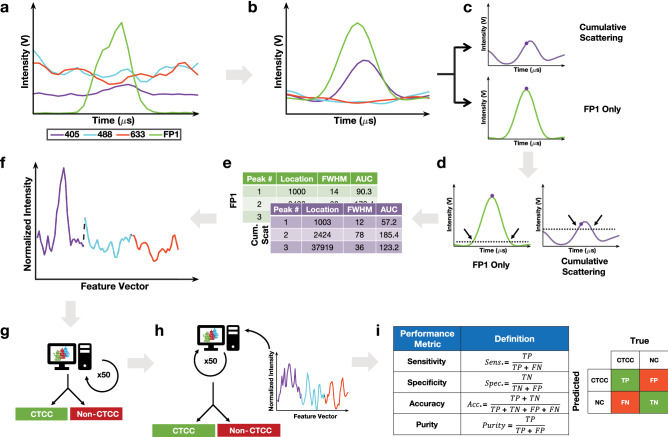
Machine learning model workflow. (**a**) Collected scattering and fluorescence data were analyzed to find the location of all cluster events. FP1 represents GFP used for ground truth labeling. (**b**) Data were initially normalized using power measurements and a second order Butterworth filter. (**c**) Data from FP1 was processed separately from the cumulative scattering data (405 + 488 + 633). The built in findpeaks.m function was used to find all local maximums in the 1.5-min data traces in both FP1 only and cumulative scattering data sets. (**d**) An intensity threshold was used to define the start and end of a peak. The threshold value was defined as being three times the standard deviation of the entire 1.5-min data trace in the FP1 and cumulative scattering channel. (**e**) Peak locations and characteristics were recorded for both the ground truth (FP1) data and the cumulative scattering data. (**f**) Using the locations of these clusters, a window of ± 13 points per scattering channel were reorganized into an 81-point feature vector. Based on FP1, we generated the labels for peaks as either being CTCC and NC events. (**g**) The generated features and labels were used to train a Gentle Adaptive Boost, Ensemble Boosted Tree classification algorithm to classify peaks. The training set included measurements from 10 days of collections while the test set was composed of 5 separate days of data. The final model was an ensemble of 50 models trained on fifty different data sets composed of the same CTCC peaks and an equal number of randomly selected NC peaks. (**h**) The test set was evaluated following training and used to classify peaks based on similarly formatted feature vectors (pseudocode can be found as Supplementary Fig. S1). (**i**) Performance metrics were calculated based on test set performance.

Such data were acquired from 18 distinct experiments and were used as training and testing datasets for the development of a machine learning model to assess the accuracy with which we can identify CTCC-associated peaks, relying on the GFP-detected peaks as the gold standard. The machine learning model was composed of two parts—the first part was an initial peak detection algorithm; the second part was a classification algorithm. During each experiment, data were acquired from ~ 135 μL of spiked blood samples, collected typically over 45 min. A second order Butterworth filter was applied to the detected scattering and fluorescence signals (Fig. 3a) to remove high frequency noise, before data from the three scattering channels was normalized based on daily power measurements and a 99% reflectance standard (Fig. 3b). Following a previously established data analysis workflow, signals from the three scattering channels were summed together, while the green fluorescence signal was processed independently (Fig. 3c)^9^. Peak locations were determined using the built-in MATLAB function findpeaks.m in the same step. An intensity threshold (3σ) was used to define peak event ranges based on where the cumulative scattering and FP1 only traces crossed the threshold value (Fig. 3d). Each peak range was inspected to determine if multiple peaks were located within a single range. If multiple peak events were found within the same peak range, only the maximum peak was saved; all other peaks in the range were deleted. Any peak that failed to cross the 3σ threshold was also deleted and assumed to be a noise event. The peak detection algorithm extracted characteristics from the remaining peaks such as peak width, area-under-the-curve, location, and intensity (Fig. 3e). While all these peak metrics were calculated and stored, for the studies discussed here, only peak width and location were used. This marked the end of the peak detection algorithm. Prior to implementing the classification algorithm, we calculated the detection purity of the peak detection algorithm to determine if additional processing was necessary. The peak detection algorithm demonstrated a purity value < 1%, leading us to explore alternative methods to further reduce the number of false positive events. To accomplish this, we examined multiple machine learning based models to optimize the detection of CTCCs while minimizing false positive peak detection.

Feature vectors for the classification algorithm were developed based on peak locations determined by the peak detection algorithm. Peaks with widths less than 20 points were removed as they could potentially originate from large single cells or WBCs. This threshold also included a majority of two cell events, which we could not reliably sort from large single cell events. As such, results for classification are focused on clusters greater than 2 cells in size. Locations from the FP1 only channel were used to generate feature vectors for the CTCC events. Peak locations in the cumulative scattering channel that did not have a corresponding green fluorescence event were used to generate the feature vectors for the NC events. In each feature vector, raw data were normalized (see “Model selection”) before features were selected for the model (see “Examination of data normalization techniques”). Feature vectors were composed of various combinations of scattering channel data. For each scattering channel, ± 13 points from the peak location were set aside for the feature vector. For example, when the three scattering channels were included in the feature vector, features 1–27 corresponded to data from the 405 channel, features 28–54 corresponded to data from the 488 channel, and features 55–81 corresponded to data from the 633 channel (Fig. 3f). 50 independent models were trained on fifty data sets composed of the 2285 CTCC peaks and fifty different combinations of an equal number of NC peaks selected randomly from a total of 44,964 NC peaks identified by the peak detection algorithm (Fig. 3g). After training, an unseen test set composed of similarly formatted feature vectors from five independent experiments was used to evaluate the performance of the ensemble of models. The test set was evaluated in a non-specific order by each of the 50 models (Fig. 3h). The initial test set was assessed by the first trained model with events predicted to be CTCC events, regardless of if they were actually CTCC events, being redefined as the new test set for the subsequent model; this process was repeated for all 50 trained models. Performance metrics were calculated based on the cumulative performance of the ensemble of models (Fig. 3i). Greater detail regarding the peak detection and classification algorithms can be found in “Methods: data processing” and “Methods: machine learning”. Additionally, pseudocode is available in Supplementary Fig. S1 online.

### GFP peak detection sensitivity assessment

Using known concentrations of CTCs spiked in whole blood, an estimated number of CTC events in a given time trace were calculated. The number of events detected in the GFP channel were also calculated. These values, when compared, allow for the assessment of GFP detection sensitivity and validation of GFP peak use as a ground truth signal. Over the course of five independent days an average sensitivity (± standard deviation) of 96.8 ± 3.44% for fluorescently-labeled CTCs was observed (Table 1). We note that in three of the five experiments, sensitivity was higher than 98.7%. Estimation errors of cell concentrations and inconsistent flow conditions (and thus interrogated blood volume estimates) may account for some of the observed differences, especially for the two days with lower sensitivity. We also note that CTCs are expected to have lower SNR than CTCCs primarily due to their size. Thus, this number represents a lower bound for the sensitivity with which GFP-labeled CTCCs were detected in our study. As the number and size of the spiked CTCCs can change as a result of processing and flow, it was not possible to acquire a robust sensitivity metric for CTCC GFP peak detection.

**Table 1:** Detection of GFP-associated CTCs in whole blood by the BSFC (± standard deviation).

Day #	Concentration (CTC/μL)	Volume (μL)	Expected # of CTCs	Detected # of CTCs	% error in detection
1	7.08	135	956	944	1.27
2	3.13	160	500	468	6.84
3	5.56	180	1000	997	0.30
4	8.13	124	1008	933	8.04
5	12.28	135	1657	1648	0.55
Avg	7.2 ± 3.4	146 ± 23	1024 ± 413	998 ± 421	3.4 ± 3.7

### Model selection

Three machine learning (ML) algorithms were assessed—a narrow neural network (NNN), a fine k-Nearest Neighbors (kNN) model, and an ensemble boosted tree model (EBT). These models were selected as kNN models are simple to implement and a good starting point for most machine learning problems. NNN’s are highly flexible and can provide reliable performance when provided enough data. Finally, EBT models are well known to work on complex, noisy data, combing multiple weak models to generate superior performance all together^22^. All machine learning models were trained and evaluated to determine the optimal combination of feature vectors, normalization techniques, and algorithms needed to achieve high levels of performance. Performance was calculated using four metrics: Sensitivity, Specificity, Purity, and Accuracy; these values were calculated based on established formulas described in detail in “Methods: metrics” (Fig. 3i). Models were trained on a data set collected during ten experiments from a total of 13 experiments and tested on a separate data set from five experiments. The average performance of all three models demonstrated that the EBT model provided the strongest classification (Table 2). Closer examination of these models supports this result as kNN and NNN models are known to overfit noisy datasets, and, as such, performance suffers^22^. The EBT model likely outperformed the other models on this dataset because of its ability to combine multiple weak models and classify events based on a consensus of multiple models^22^. This limited the effects of overfitting and allowed the model to learn features of CTCCs that were difficult to discern from NC events and classify both types of events accurately. Thus, the EBT model was used in further steps.

**Table 2:** Average performance (± standard deviation) of three ML classification models. All models were trained and tested based on the described pseudocode (see Supplementary Fig. S1) with the only variation being the classification model used.

ML model	Mean purity	Mean specificity	Mean sensitivity	Mean accuracy
NNN	95.2 ± 0.8	99.3 ± 0.1	64.9 ± 5.3	93.4 ± 0.9
Fine kNN	88.1 ± 1.8	98.1 ± 0.4	65.1 ± 4.3	92.4 ± 0.7
EBT	82.8 ± 2.1	95.9 ± 0.7	92.5 ± 2.3	95.4 ± 0.3

### Examination of data normalization techniques

**Figure 4:**
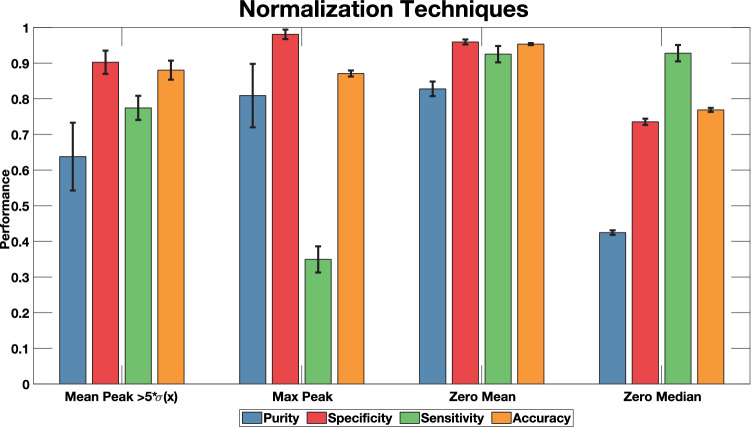
Using an identical experimental setup as the one described in Table 2, four normalization methods were applied to the dataset. However, a key difference is the classification algorithm (EBT) was fixed with the only varying variable being the normalization techniques. The Zero Mean method demonstrated consistently higher performance across all metrics indicating it is best suited for our datasets. Error bars represent one standard deviation across the 286 ensemble of models. Pseudocode of the applied method can be found as Supplementary Fig. S1.

A crucial step towards ensuring uniform analysis of data collected on multiple days was ensuring all data were normalized appropriately. To develop a model that could be potentially implemented independently of the illumination power values which varied from day to day, we sought a new normalization technique. Four methods were examined to normalize each subset of 1.5 min of data—normalizing peak intensity against maximum peak (Max Peak), normalizing against the average intensity of all peaks (Mean Peak > 5σ(x)), normalizing a zero-mean dataset by the standard deviation of all raw data (Zero Mean), and normalizing a zero median dataset by the standard deviation of all raw data (Zero Median). Each normalization technique was implemented prior to training the EBT model and then before testing its performance with a distinct data set. The same sets of data were used for training and testing when evaluating the impact of each normalization method. The optimized hyperparameters for the EBT model included 102 splits, with 84 learning cycles, and a learning rate of 0.899. Gentle adaptive boosting was also used to maximize performance on the noisy data. Results from these studies indicated that normalizing the data by subtracting the mean signal and then dividing by the standard deviation of the signal (Zero Mean) led to the overall best performance, corresponding to a sensitivity of 92.5%, specificity of 95.9%, purity of 82.8%, and accuracy of 95.4% (Fig. 4). This supported previous results we obtained when training and testing different normalization techniques on the NNN and kNN models, where the Zero Mean method also provided the best overall performance (data not shown).

### Feature space selection

Using correlation and SNR measurements, we observed the scattering signal intensity from CTCCs originated primarily from the 405 nm light scattering channel. Localized traces showed that locations with CTCC peaks had strong 405 nm light scatter (Fig. 2c–e), while random scattering events did not typically have a strong 405 scattering signal (Fig. 2f). This led us to vary the inclusion of other scattering signals to determine if they introduced noise to the analysis or benefit it. Multiple models were trained to include and exclude different combinations of scattering signals to find the optimal combination of signals needed to improve performance. Interestingly, we observed minimal differences between the models using data from the 405 and 488 nm channels (Purity: 82.58%, Specificity: 96.12%, Sensitivity: 87.58%, and Accuracy: 94.64%), the 488 and 633 nm channels (Purity: 83.56%, Specificity: 96.20%, Sensitivity: 91.23%, and Accuracy: 95.34%), and all three scattering channels (Purity: 82.78%, Specificity: 95.94%, Sensitivity: 92.51%, and Accuracy: 95.35%) (Table 3). This suggested that the model only needed two out of the three scattering channels to accurately sort CTCC from NC scattering events. To maximize detection sensitivity, we determined the optimal feature space to use for the model would combine all three scattering channels.

**Table 3:** Feature vectors examined along with average performance (± standard deviation). All models were trained and tested based on the described pseudocode (see Supplementary Fig. S1) with a fixed EBT model and zero-mean normalization method. The only variation introduced was the features used to develop the model.

Features included	Mean purity	Mean specificity	Mean sensitivity	Mean accuracy
405 ONLY	79.5 ± 3.8	97.8 ± 0.5	40.3 ± 3.0	87.8 ± 0.6
405 + 488	82.6 ± 1.4	96.1 ± 0.5	87.6 ± 2.0	94.6 ± 0.3
405 + 633	79.0 ± 2.1	95.0 ± 0.8	90.2 ± 3.3	94.1 ± 0.5
488 + 633	83.6 ± 2.6	96.2 ± 0.9	91.2 ± 2.4	95.3 ± 0.4
405 + 488 + 633	82.8 ± 2.1	95.9 ± 0.8	92.5 ± 2.3	95.4 ± 0.3

### Examining final model performance

**Figure 5:**
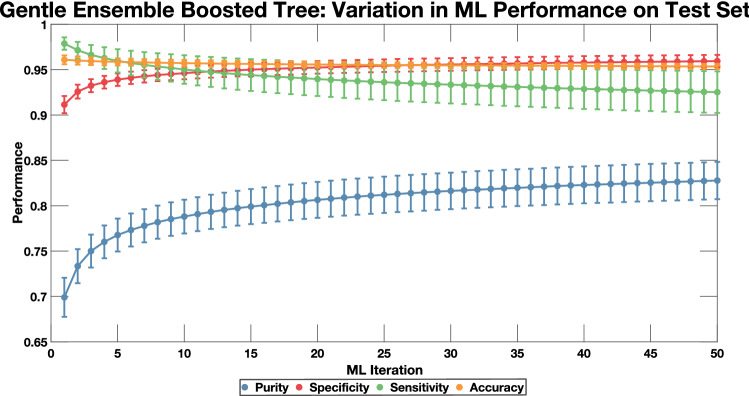
An ensemble of models were trained for classification based on data from 10 days out of a total of 13 days of data. The testing set featured 5 days of unseen data which was formatted identically to the training set. The test set was evaluated in a non-specific sequence with the predictions of first model dictating the inputs into the next model until all 50 models had evaluated the data. As we increased the number of models used, we observed a convergence in performance to a maximal limit. Error bars represent one standard deviation across the 286 ensemble of models.

To validate that we had successfully learned the features of the CTCCs, we further retrained the ensemble of models using different combinations of independent experimental datasets, generating 286 training sets. All 286 training sets were used to train an ensemble of 50 models before the test set was evaluated. The final performance values from all 286 combinations of trained models were averaged and reported (Pseudocode is provided in Supplementary Fig. S1 along with line-by-line discussion in Supplementary Discussion 1 online). The testing set was composed of 4091 CTCC events and 19,534 NC events acquired from five distinct experiments. All data were processed and normalized identically to the training set. The testing set was fed into the trained ensemble of models to evaluate sensitivity, specificity, purity, and accuracy. To this end, the average performance (± standard deviation) across all trained classification algorithms yielded a sensitivity of 92.51 ± 2.29%, specificity of 95.94 ± 0.69%, purity of 82.78 ± 2.05%, and accuracy of 95.35 ± 0.28% (Fig. 5).

## Discussion and conclusions

In vitro microfluidic cell sorting has improved the isolation of CTCs and CTCCs from whole blood samples. At the forefront of cell sorting microfluidic chips are the DLD chip and NISA-XL chip^13,14^. The DLD chip capitalized on the size and asymmetry of CTCCs to isolate them from whole blood samples. This method showed a sorting sensitivity of 98.7% for clusters greater than 9 cells in size and a sorting sensitivity of 65.5% for clusters ranging from 2 to 9 cells in size^14^. Overall, the sorting sensitivity of the DLD chip was found to be 66.7% for all sizes of CTCCs. While the DLD chip was efficient at sorting larger clusters, limitations in sorting of smaller clusters impact its diagnostic potential. To the best of our knowledge, no study has demonstrated a correlation between cluster size and metastatic potential, as such, detection of all CTCCs in a blood sample is important.

The NISA-XL chip sought to address this limitation of the DLD chip by using inertial separation to isolate clusters, with an emphasis on smaller clusters (2–3 cells in size)^13^. Edd et al. demonstrated a sorting sensitivity of 84% for clusters consisting of 2–3 cells. The authors implied the NISA-XL can deliver comparable, if not, superior sensitivity for larger clusters but no studies have been conducted to date. Additionally, sorted samples demonstrated a poor sorting purity of 5.5% with most of the sorted sample being a mixture of WBCs and RBCs. A limitation of the DLD and NISA-XL chips remains their reliance on limited volumes of blood isolated via venipuncture at distinct time points, potentially leading to over or under estimation of CTCCs present. Additionally, shear stress experienced within a microfluidic channel can impact cell cluster viability and disaggregation^23^. Optical interrogation may alleviate the shear stresses experienced by CTCCs, allowing for isolation of intact, live clusters.

Using the principles of flow cytometry, Nitta et al. and Isozaki et al. described a custom intelligent image-activated cell sorter (iIACS) which combines high throughput cell sorting, deep learning methods, and imaging flow cytometry for real time identification of rare cellular events using both FITC-conjugated concanavalin A-labeled and unlabeled samples^24,25^. The iIACS system’s original classification algorithm was built around a convolutional neural network which could sort ~ 100 events per s^24^. Using this platform, Nitta et al*.* sorted unstained platelet aggregates in 100 μL of fixed, hemolyzed whole blood with a specificity of 99%, sensitivity of 82%, and a high sorting purity of 79.5%^24^. While these results are from sorting platelets, leukocytes, and platelet aggregates, this study suggests that a similar approach could be used to sort CTCs and CTCCs from other whole blood cell components. Further advances and improvements to the iIACS system and sorting algorithm has increased the sorting rate up to ~ 2000 events per second, which suggests a 20 fold increase in blood volume processing (up to 2 mL of blood was possible based on the platelet aggregate sorting studies)^25^. In the most recent studies, Isozaki et al. demonstrated with budding yeast cells labeled with FITC-conjugated concanavalin A, a sorting sensitivity up to 96.3%. The high throughput and sorting sensitivity/specificity of the iIACS demonstrated the potential of applying deep learning methods for cell identification and sorting. However, while these studies have demonstrated sorting cluster like events, to the best of our knowledge, no reports on the sorting performance of CTCs and CTCCs in whole blood have been made. Additionally, the design of the iIACS is complex, requiring multiple experts spanning a range of fields from optics to electronics to flow cytometry, increasing the difficulty for a clinician to use the system^26^.

Advances in IVFC have opened the door for new, highly specific, and sensitive detection of rare circulating events such as CTCCs. Current advances in the field have focused on fluorescently labeled (DiD, CellTrace™ Far Red, and GFP) CTCs limiting clinical translation of IVFC^15,17,19,20^. Initial IVFC studies were performed with DiD-labeled blood and cancer cells following tail vein injections to monitor circulation kinetics^15,27^. Results from these studies demonstrated a clear relationship between injected cells and number of detected events per minute when as few as 1000 cells were injected. In addition, there was a correlation between the metastatic potential of the cancer cells and their depletion kinetics. While these studies highlighted the potential of IVFC to perform dynamic measurements with high sensitivity, staining of CTCs was required and completed ex vivo prior to tail vein injection. Prior work by our group has explored use of IVFC for detection of GFP-labeled CTCs in NOD/SCID mice^19,20^. Results from these studies demonstrated detection of GFP associated events with SNR values up to 34 dB and a similar ability to monitor depletion kinetics of injected CTCs as the original studies^19^. We demonstrated that the number of GFP-labeled CTCs detected early in tumor growth is correlated to the formation of micro-metastases detected weeks later^20^. Further, these studies highlighted the high detection sensitivity of IVFC for detection of GFP-labeled CTCs. Finally, such an IVFC set-up was used in studies by Aceto et al*.* to monitor the circulatory clearance rate of DiD-labeled MDA-MB-231-LM2 cells and clusters (a lung metastatic variant of MDA-MB-231 human breast cancer cells) in NOD SCID Gamma mice^4^. Notably, CTCCs had a higher clearance rate from the blood stream when compared to single CTCs. This, along with their increased cellular viability, could potentially explain their higher metastatic potential. While fluorescence-based in vivo flow cytometers can provide important new insights regarding the role of CTCs and CTCCs in metastasis formation, without highly specific tags for labeling of tumor cells, these methods remain only useful for experimental studies in animal models.

Tan et al. alternatively proposed a diffuse light flow cytometer (DiFC) to detect CellTrace™ Far Red-labeled multiple melanoma CTCs in athymic NCr-nu/nu nude mice with a sample rate of 284 μL per min, a false alarm rate (FAR) of 0.014 per min, and average SNR of ~ 22 dB^17^. FAR is a ratio of the number of false positive events detected per unit time. The reported DiFC sample rate was two orders of magnitude greater than previous reported IVFCs, including the discussed in vitro BSFC, which have a sample rate of 0.1–3 μL per min. High sampling rates allow for greater blood volume processing and detection of rare events in shorter time windows. A temporal matching algorithm in combination with the built-in findpeaks.m function was used in this study to improve the FAR by minimizing false positives. However, key disadvantages of the DiFC include its reliance on exogenous fluorescence which limits its application to animal models and higher non-specific background signal which impacts its ability to detect weaker fluorescent cell events.

Other groups have explored the use of photoacoustic, photothermal, and spontaneous Raman scattering for label-free IVFC; however, only the photoacoustic flow cytometer (PAFC) has ever been used for clinical acquisition of data^16,21^. Specifically, Galanzha et al. demonstrated in an in vivo clinical study of 19 healthy and 28 melanoma patients that PAFC had a detection sensitivity of 62 ± 18% and a specificity of 94.74% for CTCs^21^. CTCCs were also identified in circulation by Galanzha et al. based on broader peak width and complex peak shapes that have been associated with CTCCs; however, CTCC counts and measurements of CTCC detection sensitivity or specificity were not provided. PAFC utilized the higher absorbance of melanoma cells compared to red and white bloods cells to detect melanoma CTCs and CTCCs without exogenous labeling. In addition, due to the increased penetration depth of ultrasound waves, PAFC was able to interrogate larger blood vessels (up to 1 mm in size) for acquisition of larger blood volume data. Results from this study were significant as they demonstrated highly specific detection (specificity > 94%) of melanoma cells in humans using positive contrast; the achieved sensitivity was also highly promising, given that it was primarily impacted by single CTCs, which are smaller than CTCCs. However, its application for label-free detection of CTCs/CTCCs was limited solely to melanoma cells^21^. PAFC has also been used to demonstrate in vitro and non-invasively in vivo detection of circulating blood clots, which ranged from 12 to 20 μm in size, using negative contrast from blood background for white blood cell clots and positive contrast from blood background for red blood cell clots^28–30^. Galanzha et al*.* demonstrated a similar detection of rare circulating blood clots (CBCs) in both healthy and melanoma patients and even rarer CTC-CBC aggregates in melanoma patients which demonstrated both negative and positive contrast as a result of the white blood cell CBCs and CTCs, respectively^21^. CBCs could be a potential source for the observed broader NC events detected in our scattering channels that did not have a corresponding fluorescent event.

While the results of the study discussed in this paper were from an in vitro model and solely focused on CTCCs, we demonstrated higher detection sensitivity and specificity compared to the PAFC, which reports combined detection of CTCs and CTCCs and in vitro microfluidic chip platforms along with comparable performance to the iIACS. Additionally, in comparison to the iIACS, we achieved higher levels of detection purity while using endogenous signals. Finally, utilizing backscatter signals to classify CTCCs in whole blood addresses the limitations of current fluorescence based IVFC. To the best of our knowledge, no prior study has examined the detection of CTCCs in whole blood using endogenous scattering signal. The data analysis pipeline described in this study was critical for the detection of CTCCs in whole blood based on backscatter signals.

Application of zero-mean normalization on all three scattering wavelengths, in combination with an ensemble of EBT models, yielded an average sensitivity (± standard deviation) of 92.51 ± 2.29%, specificity of 95.94 ± 0.69%, purity of 82.78 ± 2.05%, and accuracy of 95.35 ± 0.28%. These results demonstrated the presence of unique light scattering signatures of CTCCs as the model reliably distinguishes CTCC events from NC events, independent of which combination of experimental days were used to train the ensemble of models. We also note that training with data from only two scattering channels yielded comparable levels of performance as the three scattering channels on the test set. This is beneficial for development of potentially simpler and cheaper systems, improving the potential for clinical translation.

A key limitation of the current BSFC implementation is the limited throughput of the system. In vitro processing of whole blood samples from a patient (usually 7.5 mL in volume) would take an excess of 40 h based on the described flow rates. Multichannel detection could improve processing times by allowing for simultaneous interrogation. Application of a multichannel detection system comes with increased cost and complexity for detection of signal from multiple channels. Large channels with higher flow rates could also improve throughput. However, to keep the flow parameters relevant to potential in vivo imaging targets of non-invasive, label-free BSFC, such changes would likely yield prohibitively low SNR. Thus, development of multi-capillary detection will likely be needed to provide clinically useful measurements in a reasonable time in vivo. As further development of label-free BSFC occurs, we plan to investigate such methods for improving throughput. In its current form, interrogation of superficial vessels in nailfold, volar forearm, gingival cavity, and eyes would likely be the best potential targets for the system in a clinical setting.

To achieve real-time monitoring, we need to optimize the algorithm for predicting CTCCs from raw data without the need for exogenous labels. In our current studies, green fluorescence was used to determine the location of CTCCs and NCs. This requires time processing the data and generating feature vectors for CTCCs and NCs. However, to achieve fully label-free detection of CTCCs, we ultimately need to demonstrate the algorithm’s ability to find CTCC peaks without being instructed where to look. This step is important for in vivo and in vitro studies as label-free detection of CTCCs requires no exogenous fluorescence. To this end, this paper serves as an important first step in demonstrating the CTCCs have unique detectable scattering signatures in whole blood. To develop a label-free, real-time detection algorithm, we will continue to build on the data set of CTCC events and explore deep learning techniques to further learn and identify the features corresponding to CTCCs in whole blood. In addition, we will consider improved approaches for the combination of the light scattering signals from multiple channels for peak identification. In some cases, there is a time shift in the peak locations of the traces from different wavelengths, which likely impacts adversely the overall SNR of the summed intensity traces we currently use in our data processing workflow.

Additional work will focus on improving the current model by shifting to a more versatile program for greater optimization. Currently, our implementation relies on all events having the same feature vector length. This can have limitations as clusters can range in size; a larger cluster is cut off when included in the model as we are only using 27 points from a cluster event to make a prediction. To overcome this, we need to be able to vary the feature vectors based on a cluster event size. In this way, the algorithm is not predicting based on a small portion of a cluster, but the entire cluster. Additionally, more complex algorithms and hyperparameter options exist in programs using the Python language compared to MATLAB allowing for further improvement in model performance. Using such a model, we anticipate to be able to generate feature vectors and classify peaks rapidly for real-time detection of CTCCs in whole blood.

In these studies, a high-performance cluster computer was used to train and test all data. The total runtime for a single ensemble of 50 models to predict cluster locations was estimated as less than 20 s. However, this run time was based on formatting of the desired feature vectors prior to making predictions. In the algorithm’s current form, real-time implementation would not be possible. Future improvements to the peak detection algorithm in conjunction with deep learning models could provide rapid classifications, similar in scale to what has previously been reported for the iIACS of less than 32 ms^24^.

In summary, this study demonstrated that backscatter flow cytometry provides a new potentially powerful method for label free detection and monitoring of CTCCs in whole blood. The use of machine learning approaches was critical in our ability to identify features that can be used to identify CTCC peaks based on scattering with very high accuracy. Backscatter flow cytometry for CTCC detection is currently limited to in vitro studies, but as we develop new algorithms and grow the training and testing sets, we plan to present a more comprehensive model for label-free detection of CTCCs in an in vivo animal model to demonstrate BSFC’s capability for clinical use. Even in the context of in vitro CTCC detection, label-free BSFC may offer a useful modality for CTCC isolation requiring minimal processing and yielding CTCCs with genomic and proteomic profiles that represent more accurately their state while circulating in vivo.

## Methods

### Sample preparation

Blood from healthy non-tumor bearing, non-experimentally manipulated mice and rats from other studies was drawn using cardiac puncture immediately following euthanasia via CO_2_ inhalation. 500 μL of whole blood was collected in K2EDTA BD Microtainer blood collection tubes. All animal procedures were done in accordance with the Institutional Animal Care and Use Committee (IACUC) at Tufts University and Animal Research: Reporting of In Vivo Experiments (ARRIVE) guidelines. Using non-experimentally manipulated rodents slated for euthanasia from other studies reduced the need to obtain new animals for this study. All collected blood samples were processed within 24 h of drawing. Samples were stored at room temperature until they were flowed through microfluidic channels.

Cells clusters were generated from a known and well characterized human triple negative metastatic breast cancer line, MDA-MB-231, using an established protocol for cell cluster generation^14^. Briefly, cells were plated on a standard 10 cm dish and allowed to reach 90% confluency. Once confluency was achieved, cells were lifted into solution using 1.5 mL of 0.25% trypsin (Gibco). During the process, detached cells interacted with one another generating cell clusters. Excess trypsin was deactivated using 8.5 mL of fully prepared media (10% FBS and 1% penicillin–streptomycin) after 3–5 min. Clusters were gently transferred into a 1.5 mL Eppendorf tube; care was taken to minimize mechanical dissociation of clusters. In studies focused on assessing green fluorescence peak detection sensitivity, clusters were mechanically dissociated by pipetting the sample up and down to yield single cells. 100 μL of tumor cell clusters or 300 μL of CTCs were spiked into a tube of whole blood containing 500 μL of blood. The sample was then placed on a tube rotator (VWR Tube Rotator) to mix the clusters/CTCs gently into the blood for 2–5 min. For the studies described in this paper, the mean distributions of cluster sizes and CTC concentrations were visually counted using a standard fluorescence microscope (Nikon Eclipse T*i*2). Over the course of 18 days, the average distribution of clusters sizes (± standard deviation) was found to be 65.8 ± 6.5% single cells, 17.1 ± 6.5% 2 cell clusters, 15.0 ± 4.9% 3–6 cell clusters, 1.4 ± 1.1% 7–9 cell clusters, and 0.7 ± 0.9% 9+ cell clusters. Mixed samples were flowed through a 30 × 30 µm^2^ microfluidic channel made of polydimethylsiloxane (PDMS) bonded to a glass microscope slide, as previously described^9,31^. Flexible tubing on one end of the device was connected to a reservoir to collect the flowed samples, tubing on the other end of the device was connected to a syringe containing the mixed sample. The syringe was placed on a syringe pump (Harvard Apparatus) set to push the sample at a flow rate of 3 μL/min. Channels were pre-wetted by manually injecting phosphate buffered saline (PBS). Samples were flowed for up to two hours or until the sample was completely used. A sample image of cells flowing in cell growth media through the microfluidic channel is included in Supplementary Fig. S2 (online). All experimental studies were approved by the Tufts University Institutional Biosafety Committee.

### Flow cytometer and data collection

For the BSFC setup, a 20 mW 405 nm laser diode assembly (56-ICS-425; Melles Griot), 20 mW 488 nm diode-pumped solid-state laser (PC13589; Spectra Physics), and a 20 mW 633 nm HeNe laser (1144P; JDS Uniphase) were used. The 405 nm laser was poorly collimated so a telescope was setup to collimate the beam. The 405 nm laser was first directed towards a f = 35 mm plano-covex lens (L1; LA1027-A; Thorlabs) followed by a 100 μm pinhole (Pinhole; P100S; Thorlabs), and then finally a second f = 35 mm plano-convex lens (L2; LA1027-A; Thorlabs). After careful alignment of the telescoping lens, it was confirmed that the 405 beam was collimated using a beam propagation analyzer (Modemaster M2; Coherent Inc.). All beams were directed towards mirrors to redirect the beams orthogonally (M1-4; BB1-E02; Thorlabs). In the illumination paths of the 405 nm and 488 nm lasers, neutral density (ND) filters (ND1 = 0.2 and ND2 = 0.8; Thorlabs) were used to reduce the power delivered to the sample from these lasers; an additional ND filter (ND3 = 0.5; Thorlabs) was used to reduce the combined 488 nm and 633 nm laser power. Dichroic filters (D1; 620DCXXR and D2; 465DCXR; Chroma) were used to combine the 488 nm and 633 nm lasers and the 405, 488, and 633 nm lasers together; respectively. All beams were carefully aligned to be colinear. A linear polarizer (Pol1; 03FPG021; Melles Griot) was used to control the polarization of input light from the colinear 405, 488, and 633 nm laser light. Vertically polarized light was directed towards a f = 150 mm MgF_2_ cylindrical lens (Cyl Lens; NT48-367; Edmund Optics) to form a horizontal slit. This horizontal slit was refocused 300 mm from the Cyl Lens by a f = 150 mm achromatic lens (L3; NT32-494; Edmund Optics) to infinity. A 50:50 beamsplitter (BS1; Chroma) was placed after L3 for later use in detection and for verification of alignment by projecting the input beam off a mirror (M5; Thorlabs) to a faraway screen. Transmitted light was reflected off a mirror (M6; Thorlabs) secured on a 45°-degree mount towards the sample stage. M6’s mount was secured to a single-axis translation stage with a standard micrometer (Thorlabs) to vary the illumination and detection angle between 0° and 18°. A filter cube was situated above M6 to hold a 40×, NA = 0.6 objective (LUCPLFLN; Olympus) and internally another 50:50 beamsplitter (BS2; Chroma) for transillumination. For all studies, a xyz-translation stage was built around the objective to position the sample across the slit and set the focal depth of collection. All power measurements were taken at the sample stage used a photodiode Si sensor (PM16-120; Thorlabs). The position of samples was verified using transillumination by a green LED (Luxen V), whose transmitted light reached BS2 through the objective, and reflected to form an image of the device channel and illumination slit on a CCD camera (Watec) through a f = 150 mm achromatic lens (L4; Edmund Optics). Backscattered light from the channel was collected through the same objective. A beam block was secured to a secondary micrometer and placed immediately below the filter cube. The beam block was advanced to restrict the collection of backscatter signal between 0° and 18° (identical to the illumination beam) and block specular reflection. This was confirmed by imaging the illumination slit as we adjusted the beam block position until we observed clipping of the slit. This process was carried out in order to improve the SNR of the data by reducing the background intensity from glass reflections. Backscattered and fluorescence light that followed the illumination path were focused onto a 150 × 3000 μm slit aperture (Confocal Slit; S15OR; Thorlabs) using a f = 150 mm achromatic lens (L5; Edmund Optics). Focused backscattered and fluorescence light exited the slit and was refocused using another f = 150 mm achromatic lens (L6; Edmund Optics) to infinity while unfocused light was blocked. The polarization of the detected light was ascertained by a second linear polarizer (Pol2; Melles Griot) that was set to be colinear with Pol1. Finally, signals were separated using dichroic mirrors (D3-D6; Chroma) and directed toward five photomultiplier tubes (PMTs). The first dichroic mirror (D3; 460DCXRU; Chroma) was used to isolate the 405 nm signal to PMT1. The second dichroic mirror (D4; 629DXR; Chroma) was used to isolate the 633 nm and deep red fluorescence signal from the 488 nm and green fluorescence signal. The third dichroic mirror (D5; 500DXR; Chroma) was used to split the 488 nm signal from the green fluorescence signal, delivering each to PMT2 and PMT3, respectively. Finally, the fourth dichroic mirror (D6; ZT647rdc; Chroma) was used to split the 633 nm signal from the deep red fluorescence, sending the signal to PMT5 and PMT4, respectively. A bandpass filter (Chroma) was placed in front of each PMT (R3896; Hamamatsu) to collect scattered light in the ranges of 405 ± 5 nm (BP1; Z405/10×), 488 ± 5 nm (BP2; Z488/10×), and 633 ± 5 nm (BP5; Z633/10×), and fluorescence in the 500–550 nm (BP3) range and 650–690 nm (BP4) range. Additionally, due to signal saturating the PMTs for the three scattering wavelengths (PMT1, PMT2, PMT5), ND filters (ND4 = 0.6, ND5 = 1.2, ND6 = 0.9; Thorlabs) were added before each PMT, respectively, to ensure we could collect high SNR data without damaging the detectors.

### Data processing

Data were sampled at 60 kHz and digitally recorded using a data acquisition unit (USB-6341; National Instruments) into LabVIEW (v18.0; National Instruments) before being transferred into MATLAB. Raw data were read in 1.5-min data increments to ensure drifts in baseline signal could be readily normalized. A second order Butterworth filter was used to remove high frequency noise and normalize the baseline signal (50–6000 Hz). After filtering, signal intensities from the three scattering wavelengths (405 nm, 488 nm, and 633 nm) were normalized based on incident power on the sample ($Powe{r}_{\lambda ,Sample}$), reduced incident power on the spectralon ($Powe{r}_{\lambda ,Spectralon}$), and corresponding intensities backscattered from a 99% spectralon using the reduced power ($Intensit{y}_{\lambda , Spectralon}$). Power was reduced using a ND filter with an OD = 1.0. The normalization factor was calculated daily for each of the scattering wavelengths using Eq. (1):

1 $$Norm\left(\lambda \right)=Intensit{y}_{\lambda , Spectralon}\cdot \frac{Powe{r}_{\lambda ,Sample}}{Powe{r}_{\lambda ,Spectralon}}, where \lambda = 405, 488, or \; 633 \; nm$$

Signals from the three scattering channels were summed into a single cumulative signal (405 nm signal + 488 nm signal + 633 nm signal). The sum of all three scattering signals improved the SNR for peak detection, with spurious peaks averaged out^9^. The cumulative signal of each 1.5 min segment was analyzed using the built in MATLAB function findpeaks.m in the Signal Processing Toolbox to identify peaks. As a ground truth comparison to the scattering channel data, green fluorescence peak locations were also interrogated using the same algorithm. The premise of this step was to use the ground truth signal (green fluorescence) to identify the locations in the cumulative scattering channel that corresponded to CTCCs. To label peak events, an intensity threshold of 3σ was applied to both the cumulative scattering and green fluorescence channel. Peaks that failed to meet this threshold were removed from further processing. A 5σ threshold was also explored for the green fluorescence channel but yielded identical peaks. Data from FP2 (red fluorescence) channel were also collected in order to determine if endogenous red fluorescence signatures from CTCCs were present. However, further examination of the red fluorescence signal revealed crosstalk from the GFP label; as such, FP2 was omitted from all analysis.

Once the location of peaks in both the cumulative scattering and green fluorescence channels were identified, peak characteristics were extracted and saved. The full width at half maximum (FWHM), scattering intensity of each of the five acquisition channels at the specified location, the area-under-the-curve (AUC), cumulative scattering intensity, and FWHM and AUC of peaks within the five individual acquisition channels were calculated. These values along with location of the peak were stored for further analysis using the classification algorithm. For the discussed studies, only the FWHM and peak locations were used. Other values were stored for potential further analysis and/or model development. Figure 3 provides a general overview of the data processing steps used.

### Assessment of GFP sensitivity detection

GFP labeled CTCs were used to validate the sensitivity of GFP peak detection when flowing cells in whole blood. CTC concentrations were measured during five independent experimental days before a fixed concentration was spiked into whole blood samples. As samples were flowed at a fixed flow rate, estimates of blood volume interrogated and concentration of CTCs in blood were used to predict the number of CTC events that should be present in the green fluorescence data. The expected counts and actual number of GFP peaks detected using our peak detection workflow were compared. Expected # of CTCs were calculated by multiplying the concentration by the volume flowed. Detected # of CTCs were calculated by counting the number of fluorescent peaks in the green fluorescence data channel for the same estimated flow volume.

### Machine learning

Three ML models were assessed—a NNN, a Fine kNN model, and an EBT model. All ML models were trained and evaluated to determine the optimal combination of feature vectors, normalization techniques, and algorithms needed to achieve high levels of performance. All pre-processing of data were completed in MATLAB R2021a with four different normalization techniques being applied to generate four master sets of feature vectors for all peaks. As described above, using the peak locations defined by the peak detection code, we normalized each subset of 1.5 min of data in four different ways –Max Peak, Mean Peak > 5σ(x), Zero Mean, and Zero Median. After the data were normalized, for each method, we selected ± 13 points on either side of each peak from the three scattering channels. The center of the peak was selected based on the output of peak locations in the cumulative scattering and green fluorescence channel from the peak detection algorithm. This allowed for a holistic determination of peak center for the three scattering channels as the maximum peak location could vary per wavelength. The selection of ± 13 points was based on the expected minimum FWHM of a cluster event being 20 points allowing us to ensure the full range of points for the smallest CTCCs was included. We were able to calculate the minimum FWHM of a cluster event based on the sample flow rate and cross-sectional area of the channel which gave us an estimate of the flow velocity. Using the expression v = Q/A, where Q is the sample flow rate, A is cross sectional area, and v is the flow velocity, we determined that the flow velocity was 55.6 mm/sec (Q = 3 μL/min and A = 900 μm^2^). Next, we estimated the time for a large CTC or WBC (~ 12–15 μm) to cross the illumination slit which had a width of 5 μm. This was calculated by the expression Δt = d_eff_/v, where Δt is the time, d_eff_ is the effective diameter of the expected event, and v is the flow velocity. Using the largest single cell size expected (15 μm), we calculated Δt to be 3.6e − 4 s (d_eff_ = 15 + 5 = 20 μm and v = 55.6 mm/s). With a sample rate of 60,000 samples per second, this corresponded to a width of ~ 21 points, as such we anticipated events ≥ 20 points in width to be a cluster event. Two cell clusters presented a unique challenge for detection as their orientation when crossing the illumination slit could affect the size of the detected peak. Thus, we could not reliably identify two cell clusters from single cells and report algorithm performance for clusters of 3 cells or larger in size. This also means for small clusters, a window of ± 13 points will capture the full cluster. The three sets of 27 points of data from each scattering channel were finally organized as a vector of features (405 nm channel = features 1–27, 488 nm channel = features 28–54, 633 nm channel = features 55–81). This process was repeated for all normalization methods. Peak locations corresponding to single cells were removed before further processing as we were solely interested in CTCCs. To remove CTC events, a simple threshold was used to remove detected events with FWHM’s less than 20 points in size (corresponding to a cell size of ~ 14 μm, see calculation above). This value was selected based on the maximum size of both white blood cells and CTCs we expected to see in the sample. The remaining events were labeled as being potentially CTCCs. The total number of CTCC events detected per day was determined from the green fluorescence channel (FP1) which served as the ground truth (see Supplementary Table S1 online). As only the CTCCs exhibit green fluorescence, we classified any detected scattering event that did not show a corresponding peak in the FP1 channel as a NC scattering event. CTCC feature vectors were developed for all events detected in the FP1 channel. NC feature vectors were taken from the cumulative scattering data by extracting all locations where there was no green fluorescence peak present (Fig. 2F). From a total of 18 days of experimental data available, data from 13 days was set aside for training and data from 5 days was set aside for testing. During training, 10 days of data from the total of 13 days was used in training with the remaining ~ 25% used for validation. Validation sets were used to ensure the model was successfully trained. To improve on processing time, we utilized the Tufts High Performance Cluster, where a single 8 core CPU with 50 GB of memory was allocated for all computations. On the cluster, the master feature vector set was uploaded for all normalization techniques along with scripts for three different machine learning algorithms written by the built in Statistics & Machine Learning toolbox in MATLAB R2021a. Finally, an evaluation script was uploaded to train 286 combinations of 10 experimental days of training data out of 13 possible days before being tested on an unseen 5 experimental days of testing data (pseudocode can be found in Supplementary Fig. S1 online). Average testing performance was calculated from all 286 different combinations of training days and reported with standard deviation in performance (Fig. 5). Several combinations of peak features from different scattering wavelengths were assessed by the machine learning model to identify the feature combination that yielded optimal CTCC detection. For example, if only the 405-channel data were desired, we selected columns 1–27 and column 82 which contained the peak labels for CTCCs and NCs. For any trained model, the maximum number of true CTCC cases was 2285 events with a total number of 44,964 NC peaks. These values were calculated by summing together the number of events present in a training set that were labeled as CTCCs and NCs based on their signal in FP1. An ensemble of 50 models was used to minimize bias due to the disparity in the number of NC peaks to CTCC peaks. To generate the ensemble of models, we started by training 50 random models using the same CTCC events and an equal number of randomly selected NC peaks. This ensured that the model learned a wider range of features from the NC peaks while maintaining its ability to detect CTCC events. The trained ensemble of 50 models was used to evaluate the test set. Using a non-specific sequence, we fed the test set into one model at a time and collected all events predicted to be CTCCs. Using only the predicted CTCC events, we called the next model to make a new prediction on these events. This process was conducted repetitively until all 50 models were exhausted. The overall classification performance after all 50 models was tabulated by summing together the total number of NC peaks correctly predicted as NC peaks and the total number of CTCCs incorrectly predicted as NC peaks to determine the final model performance. This process was repeated for all the 286 ensemble of models with performance being tracked each time a new model was introduced. Pseudocode is provided in Supplementary Fig. S1 online with a line-by-line explanation of this process (See Supplementary Discussion S1 online).

### Metrics

To evaluate a model’s classification performance, four metrics were examined—Purity (also referred to as precision), Sensitivity, Specificity, and Accuracy defined as:

2 $$Purity=\frac{True \; Positive}{True \; Positive+False \; Positive}$$

3 $$Sensitivity=\frac{True \; Positive}{True \; Positive+False \; Negative}$$

4 $$Specificity= \frac{True \; Negative}{True \; Negative+False \; Positive}$$

5 $$Accuracy= \frac{True \; Positive+True \; Negative}{True \; Positive+True \; Negative+False \; Positive+False \; Negative}$$

From these metrics we assessed the model’s ability to learn the features of the CTCCs in comparison to NC events. Measured values were compared against values from existing technologies for CTCC detection. Metrics were calculated for a wide range of models, feature vectors, and normalization techniques.

## Supplementary Information

Supplementary Information.

## Data Availability

The raw datasets used for model generation in the current study along with the trained classifier and scripts (written in MATLAB) are available from the corresponding author on reasonable request.
