# Supplementary material for: Label-free flow cytometry of rare circulating tumor cell clusters in whole blood
Source: Sci Rep. 2022 Jun 24;12:10721. doi: 10.1038/s41598-022-14003-5 (PMC9232518; doi:10.1038/s41598-022-14003-5)
Supplement: Supplementary file 1 — Supplementary Information. [file 41598_2022_14003_MOESM1_ESM.pdf]

Label-free Flow Cytometry of Rare Circulating Tumor Cell Clusters in Whole Blood

Nilay Vora[1], Prashant Shekhar[2], Michael Esmail[3], Abani Patra[4], Irene Georgakoudi[1]

[1] Department of Biomedical Engineering, Tufts University, Medford, MA 02155
[2] Department of Mathematics, Embry-Riddle Aeronautical University, Daytona Beach, FL 32114
[3] Tufts Comparative Medicine Services, Tufts University, Medford, MA 02155
[4] Department of Computer Science, Tufts University, Medford, MA 02155

Supplementary Table S1: Number of CTCC events detected per experimental day.

Day	*Total Number of Events*
1	143
2	127
3	11
4	243
5	113
6	146
7	296
8	11
9	51
10	173
11	162
12	527
13	354
14	317
15	433
16	1476
17	1680
18	185
Total number of events	6449

ML Cross Validation Script

Input:

$\mathbf{X}_{TP\_training}$:Feature Vector for all CTCC events,
$\mathbf{X}_{FP\_training}$:Feature Vector for all non-CTCC events.
$\mathbf{X}_{TP\_testing}$:Feature Vector for all CTCC events,
$\mathbf{X}_{FP\_testing}$:Feature Vector for all non-CTCC events.

Output:

$\mathbf{Sensitivity}_{M50}, \mathbf{Specificity}_{M50}, \mathbf{Purity}_{M50}, \mathbf{Accuracy}_{M50}$

Function MLCrossValidation
$C_{Days} = C\binom{13}{10} \leftarrow$ Calculates all combinations of 10 days from 13 options
$loc_{SingleCell} = find(X_{TP\_training}(FWHM) \& X_{FP\_training}(FWHM)) < 20$
delete $X_{TP\_training}(loc_{SingleCell}, :) \& X_{FP\_training}(loc_{SingleCell}, :)$
for $i = 1 : length(C_{Days})$ **do**
  $X_{TP\_training}(C_{Days}(i)) \leftarrow$ All True CTCC peaks in 10 day combination
  $X_{FP\_training}(C_{Days}(i)) \leftarrow$ All Non-CTCC peaks in 10 day combination
  $TrainingData = [X_{TP\_training}; X_{FP\_training}]$
  $label = TrainingData(:, end) \leftarrow$ Last column of data is the label
  $Features = TrainingData(:, 1 : end - 1) \leftarrow$ Training Features to use
  for $j = 1 : 50$ **do** $\leftarrow$ Training Loop
    $N_{TP} = length(label == \mathbf{True}) \leftarrow$ # of CTCC events
    $N_{FP} = length(label == \mathbf{False}) \leftarrow$ # of Non-CTCC events
    $loc_{FP} = find(label == \mathbf{False}) \leftarrow$ Location of Non-CTCC events
    $loc_{TP} = find(label == \mathbf{True}) \leftarrow$ Location of CTCC events
    $Y_{training\_FP} = Features(loc_{FP}(randperm(N_{FP})(1 : N_{TP}), :))$
      $\leftarrow$ Grabs random subset of FP peaks equal to # of TP events
    $Y_{training\_TP} = Features(loc_{TP}, :) \leftarrow$ Grabs all CTCC events
    $Y_{training} = [Y_{training\_TP}; Y_{training\_FP}]$
    $Model(j) = ML\_Training\_Model(Y_{training})$
  end for
  $TestingData = [X_{TP\_testing}; X_{FP\_testing}]$
  for $k = 1 : 50$ **do** $\leftarrow$ Testing Loop
    $Testlabel = TestingData(:, end)$
    $TestFeatures = TestingData(:, 1 : end - 1)$
    $yfit = Model(k).predictFcn(TestFeatures)$
    $TestingData = [TestFeatures(find(yfit == \mathbf{True})),$
      $Testlabel(find(yfit == \mathbf{True}))]$
  end for
  $\mathbf{Sensitivity}_{M50} = \frac{TP}{TP+FN} \leftarrow$ Defined after 50 iterations
  $\mathbf{Specificity}_{M50} = \frac{TN}{FP+TN} \leftarrow$ Defined after 50 iterations
  $\mathbf{Purity}_{M50} = \frac{TP}{TP+FP} \leftarrow$ Defined after 50 iterations
  $\mathbf{Accuracy}_{M50} = \frac{TP+TN}{TP+TN+FP+FN} \leftarrow$ Defined after 50 iterations
Return $\mathbf{Sensitivity}_{M50}, \mathbf{Specificity}_{M50}, \mathbf{Purity}_{M50}, \mathbf{Accuracy}_{M50}$
end for
End

Supplementary Figure S1: Pseudocode for iterative Machine Learning model. This algorithm was used for the NNN, fine kNN, and EBT models and repeated for all normalizations techniques and feature vectors using a EBT model.

Supplementary Discussion S1: To evaluate the machine learning model (ML Cross Validation Script) discussed in the parent text, a master set of feature vectors was first generated based on the desired normalization method and feature selection. The master set of feature vectors is broken up by experimental day of collection and whether the features are from CTCCs or NCs. The algorithm starts with first computing all combinations of 10 days out of a total of 13 days (**Line 1**). This leads to 286 unique combinations of 10 days between day 1 and day 13. In **Line 2 and 3** we find the locations of all events with a FWHM <20 points and remove those feature vectors from our training set as we are only interested in CTCCs which are ≥20 points in size, a similar method is applied to the test set (not shown here). Once we have completed these initial steps, we begin to loop through the 286 combinations we previously calculated (i.e., C_{Days} (1) = [1,2,3,4,5,6,7,8,9,10]). In **Line 5 and 6** we extract all CTCC and NC events from the specified days. We combine these events into one dataset in **Line 7** before separating the labels from the features in **Line 8 and 9**. We next start a subloop to train 50 random models using the same CTCC events and a random subset of NC peaks. In **Line 11 and 12** we calculate the number of CTCC and NC events in the training set. In **Line 13 and 14** we find the locations of all CTCC and NC events in the training set. In **Line 15** we choose a random subset of the NC peak events equal to the total number of CTCC events present in the specified days of data. This step also allows us to ensure that we have an equal number of CTCC and NC events in the training set. In **Line 16 and 17** we put together the final training set and feed it into the desired machine learning model (i.e., EBT, fine kNN, NNN) in **Line 18**. The trained model is saved before this process is repeated 49 more times. As the random subset selected changes each iteration, the NC events included in training will vary from model to model. After we have trained all 50 models, we start testing in **Line 20.** Testing data is organized similarly to the training data, and we begin a new loop for testing. To begin, in **Line 22 and 23**, we grab the labels and features. We then predict on the features from **Line 23** in **Line 24** starting with the first trained model. In **Line 25 and 26** we grab all events that the first model labeled as a "True" or "CTCC" events and recreate the testing dataset as being only these events, regardless of if they are CTCC or NC events. Through this process we are aware of the true label of all events and apply this label to generate metrics of performance after each model. The new test set, as defined by **Line 25 and 26,** is again evaluated but this time by the second trained model. We again grab all events predicted to be "CTCC" events by the second model and recreate the test set. This process is repeated until all 50 trained models have made a prediction based on the output of the previous model. In **Line 28-31,** we calculate all the final metrics which are stored for the combinations of days used to train the 50 models. This process is restarted again with a new combination of 10 days being used (an example combination can be C_{Days} (2) = [1,2,3,4,5,6,7,8,9,11]). The models are again retrained and evaluated repetitively until all 286 combinations are exhausted. The final performance values from all 286 combinations are averaged and reported.

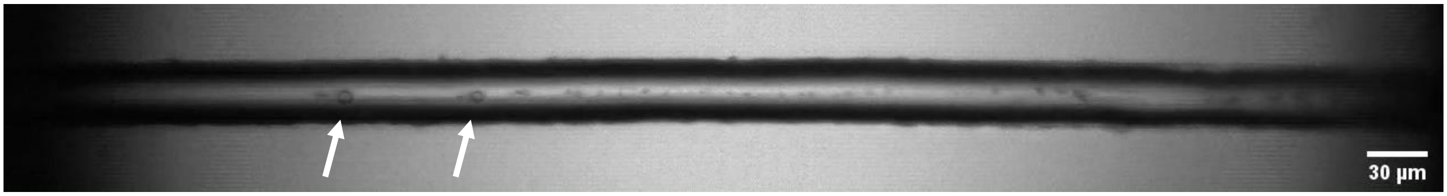

Supplementary Figure S2: An image of CTCs flowing through a 30x30 µm2 channel at 3 µL/min. CTCs are indicated by white arrows. The image was acquired using a FASTCAM Mini UX100 type 800K-M-16G at 500 fps with white light illumination of CTCs in cell growth media. Note that only CTCs were expected to be present in this flowed sample.
